# S-shaped reverse sural flap for reconstruction of tissue defect on heel

**DOI:** 10.4103/2321-3868.113334

**Published:** 2013-06-18

**Authors:** Hamid Reza Fathi, Mehdi Fathi, Mihan J. Javid

**Affiliations:** 1 Department of Anesthesiology, Imam Khomaini Medical Center, Tehran University of Medical Sciences, Tehran, Iran; 2 Department of Plastic and Reconstructive Surgery, Tehran University of Medical Sciences, Tehran, Iran; 3 Imam Khomaini Medical Center, Keshavarz Blv., Gharib St., Tehran, Iran

**Keywords:** Limb trauma, reverse sural flap, modified technique

## Abstract

Traumatic limb injury is a prevalent lesion in Iran. Motorcycle accidents are responsible for most of these traumatic lesions. Despite various reported techniques, the coverage of the Achilles tendon, malleoli, ankle and heel is still daunting and demanding procedure. S-shaped reverse sural flap is a modified technique of reconstruction. In this report of 6 patients underwent surgical reconstruction by this modified technique we discuss about the technique as a simple and safe technique with low morbidity rate and recommend using this technique in complex injuries.

## Introduction

During the recent decades in Iran, surgeons have encountered a wide variety of limb trauma among the war survivors. On the other hand, motorcycle, a commonly used vehicle in Iran nowadays, is responsible for some part of severe limb trauma. Spoke-wheel injuries commonly affect the heel region. Most of such injuries result in an avulsion flap with or without exposure of the tendoachilles and/or calcaneum. Spoke-wheel injuries seem deceptively mild initially but they usually need hospital admission, surgical intervention and a prolonged period of time for full recovery.

**Table:** 

Access this article online	
**Quick Response Code**:	**Website**: www.burnstrauma.com
	**DOI**: 10.4103/2321-3868.113334

Despite various reconstructive techniques, coverage of the Achilles tendon, malleoli, ankle and heel after loss of substance in traumatic events, especially when associated with fractures and complex wounds remains a daunting and demanding procedure. During the past two decades, pedicled flaps from the ipsilateral uninjured area of the lower leg have been developed. This large skin territory provides enough tissue source for reconstruction of the foot and ankle.[1]

After the first report on a distally based sural neurocutaneous flap for repair of injury of the lower part of the limb,[2] this flap has been modified into three types: (1) the distally based reverse-flow island flap, such as the posterior tibial artery flap, the anterior tibial artery flap, and the peroneal artery flap; (2) the distally based perforator flap in which sacrifice of the main arteries can be avoided, such as the lateral and medial supramalleolar perforator flap; and (3) the distally based neuro-veno-fasciocutaneous flap that is supplied by the chain-linked longitudinal directed vascular plexuses of the neuro-veno-adipofascial pedicle.[3–8] These flaps can be raised easily and substituted for microsurgical flaps for distal lower leg and foot and ankle reconstruction in certain circumstances. However, inferiorly-based muscle flaps are prone to some complications, including flap congestion and partial flap failure caused by inadequate venous return, complete flap failure caused by torsion or compression of the wide and thick adipofascial pedicle under subcutaneous tunnel, and donor site morbidity.[9,11]

**Figure 1:**
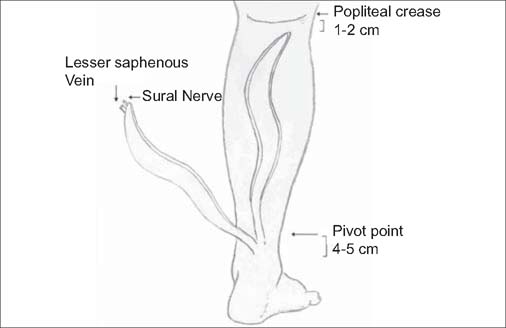
Diagram of the S-shaped reverse sural flap showing relevant applied surgical anatomy.

After a decade of our practice in applying the conventional flap design, we encountered not very favorable aesthetic results of donor site, particularly in children and women. In this context, we shared our experiences with six cases of heel defects salvaged by S-shaped reversed sural neurofasciocutaneous flaps. The goal of the current study is to redefine the flap parameters with respect to direction, pedicle, and reliability of the use of configurationally modified sural flaps in chronic or acute traumatic situations. In this new approach, we refined the surgical technique considering the safe elevation of a long flap and potential extension of its territory in the light of proper concept of neurovascular structure [Figure 1].

## Methods

From February 2005 to June 2007, a total of 6 patients with acute and/or chronic wounds of the foot and ankle underwent repair with sural flap transfers. The initial wounds were caused by spoke injury in four cases, while two others who had survived the war had pressure sore with bone and/or tendon exposure. Patient age ranged between 9 to 48 years at the time of reconstruction [Table 1]. We used an S-shaped reverse sural flap to reconstruct the heel defect. The largest flap had a length of 22.0 cm and a width of 3.5 cm. The donor area was primarily repaired in all patients.

**Table 1:** Summary of patients

Patient No.	Age/Sex	Cause of Defect	Site of defect	Size of defect (cm)	Size of Flap (cm)	Complications
1	9/F	Spoke-wheel injury	Heel	5×5	2.8×12	None
2	10/M	Spoke-wheel injury	Heel	4.5×7.5	2.5×16	None
3	13/F	Spoke-wheel injury	Heel	5×7	2.5×15	None
4	48/M	Pressure sore	Heel/Medial malleolus	4×10	2.2×22	None
5	38/M	Pressure sore	Heel	6×10	3.5×22	None
6	37/F	Spoke-wheel injury	Heel	4×9	2.5×20	None

### Flap design and surgical technique

Under general and/or regional anesthesia, the flap was approached with the patient in prone position. First, debridement of the recipient area was done and the size of the tissue defect was measured. All sural fasciocutaneous flaps were centered on the line which was drawn from the midpopliteal point to the midpoint of the Achilles tendon-lateral malleolus. We considered an S-shaped path which describes the course of the lesser saphenous vein and the superficial sural nerve. The pivot point of the flap pedicle was marked at 4 to 5 cm above the lateral malleolus in the lateral retromalleolar region, including the distal perforating branches which make anastomosis with the peroneal artery. The S-shaped flap was then outlined from this pivot point on the lower leg, as its width was thoroughly 55% of the horizontal dimension and its length was twice the vertical dimension of the recipient site. The proximal end of “S” extended well beyond the conventional confines, i.e. into the upper third of the leg, just short of the popliteal crease [Figure 1]. To facilitate flap dissection, the tourniquet was inflated without exsanguination of the involved limb.The incision was initiated in the proximal extreme of the flap. Posterior dissection was made at the plane below the deep fascia. The dissection of the S-shaped flap extended up to the demarcated limit of 4 to 5 cm above the lateral malleolus. The flap elevation proceeds distally (after dividing the nerve, artery, and lesser saphenous vein) in the subfascial plane to the perimeters of the planned flap circumferentially. The flap was rotated to reach the recipient site with no tentsion. During transposition, care was taken so as not to compress the pedicle. At this stage the longitudinal S-shaped flap is folded side by side to create a surface area compatible with the dimensions of the defect. Hemostasis was achieved with bipolar cautery after the tourniquet was deflated. In all patients, the donor site was closed primarily as a lacy *S* in two layers. Surgical time lasted from 45–70 min for flap elevation, tissue defect reconstruction and wound closure.

Postoperatively, supportive anterior plaster of Paris or an Omega-shaped plaster was used for immobilization in all patients for 2–3 weeks. After the plaster was removed, patients began an active and passive physical rehabilitation program to achieve the maximum range of motion of the repaired extremity. Patients were encouraged to walk with help at Week 2 or 3 postoperatively, and full weight-bearing was allowed in the sixth postoperative week.

## Case report

### Case 1

**Figure 2:**
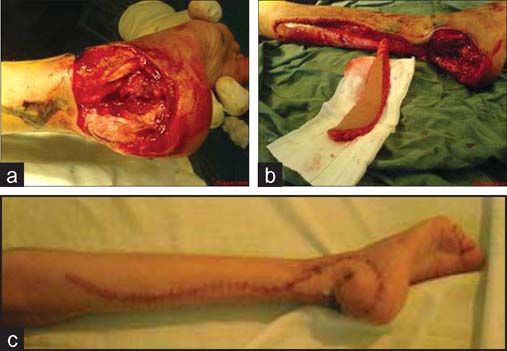
Clinical photographs in case 1. S-shaped reverse sural flap for reconstruction of tissue defect on heel. (a) Wound of the recipient region. (b) Fasciocutaneous island of sural flap was elevated. (c) Postoperative appearance with S-like scar of the donor site.

A 9-year-old girl suffered from a spoke-wheel injury with an extensive avulsion over the posterolateral aspect of her right foot and ankle and rupture of the Achilles tendon was referred to our center. She had been admitted to the orthopedic ward for 13 days. After debridement, there was a 5×6 cm fasciocutaneous defect with the exposure of bone, joint and tendons. There was no possibility of local transposition or of rotational flaps from the foot area, so the only salvaging procedure was a distal flap. The wound was covered with an S-shaped reverse sural flap that measured 2.8×12.0 cm. The donor site was repaired with direct closure and healed with no complications. The postoperative period was uneventful [Figure 2].

## Results

The first three cases and the last patient encountered spoke- wheel injury with extensive avulsion of fasciocutaneous tissue on the heel. This damage resulted in exposure of the tendons and underlying bone. Another two males who had been disabled during the war for several years developed pressure sore on the lower leg. Finally, all six flaps completely survived after surgery. Mild swelling was seen only in some flaps. Flap necrosis and venous congestion did not occur. In donor site, normal wound healing developed optimally. No complications were observed in the donor site. None of the cases needed any secondary interventions. Aesthetically and functionally pleasing results were obtained for all reconstructed tissue defects, and our patients gained satisfactory outcome [Table 1].

## Discussion

The fasciocutaneous flap from the sural region of the lower leg has been extensively investigated in respect to vascularization and the concept of neurocutaneous flaps. However, the reconstruction of heel defects remains limited and problems can occur by using these possibilities. Anatomical studies have showed a three-dimensional vascular architecture of the posterior lower leg integument from the superficial sural artery, the septocutaneous perforators, peroneal artery, and myocutaneous perforators. There is prominent longitudinal orientation or axiality of the circulation of the facial, paraneural (sural nerve), and perivenous (lesser saphenous vein) vascular plexuses. There are also 4 to 5 axial communications between this longitudinal neuro-veno-adipofascial plexus and the posterolateral septocutaneous perforators issued from the peroneal artery[12,13] With distal axial perforator perfusion, blood flow can reach a long distance in the lower-resistance longitudinal vascular plexuses and results in survival of a large flap without arterial ischemia.

Because of the anastomosis between the peroneal artery perforator and the longitudinally oriented median suprafascial sural artery, this type of flap can be based not only proximally, but also distally[1417] This flap has been designed with a long and narrow pedicle and a wide arc of rotation, because its vascular axis has the largest direct artery of the posterior calf and strongest peroneal perforator at its pivot point. This allows elevation of the skin supplied by this neurovascular axis as a flap for coverage of leg wounds, with the entire flap being axial in nature.

Recent modifications of distally based perforator sural flap overcame most of the drawbacks of the original technique. Ayyappan *et al.*,[11] introduced the super sural flap, harvesting all available tissues in the upper third of the leg, and just preserving 1 to 2 cm of skin from the popliteal crease. The length of the pedicle or size of the flap influenced neither congestion nor survival of the flap. Chang *et al.*,[18] and Zhang *et al.*,[19] reported their clinical experience on the transfer of distally based sural flap with a lower vascular pivot point. They demonstrated that the blood supply of the distally based sural neuro-veno-fasciocutaneous flap can be pivoted at lower perforators in the posterolateral region, which are about 3 and 1 cm proximal to the tip of the lateral malleolus. The greatest length of the flap in those series was 30 cm, and it survived completely without complications.

In our study, we designed a longitudinal S-shaped flap which its proximal end extended well beyond the conventional confines into the upper third of the leg. The width of “S” considered was about 55% of the horizontal dimension of the recipient site and its length was twice the vertical dimension of the defect. The additional 5% of flap area can compensate the probable shrinkage of flap after harvesting. In the current technique, the width of the flap was gradually increased from 1.5 cm to 3.5 cm. The inclusion of skin over the shorter pedicle facilitated better manipulation of the flap, increased margin of safety, avoided the dog-ear deformity, and reduced morbidity at the donor site. In this technique the flap is a type A fasciocutaneous flap which is innervated by the medial sural cutaneous nerve (s1–2), and the lateral nerve is not involved. All flaps survived with no complication and wound healing was complete at the donor site. Additionally, the pedicle of the flap was supported by the distal portion of the flap itself, so the tension on the pedicle and the pivot point was relieved.

In previous studies, the major disadvantage of reverse sural flap was an unsightly scar over the posterior calf when a fasciocutaneous flap was used. This problem can be avoided by taking only the fascia and covering it with a skin graft. The neurologic deficit caused by the sacrifice of the sural nerve is negligible. The distally based neurocutaneous sural flap is an excellent choice in the pediatric age group for covering defects of the lower leg and foot, where thin pliable skin is needed. The aesthetic outcome of the flap transfer might be unsatisfactory, especially in females and children.

In our modifications, this shape of the flap relieved the tension on the suture line and prevented the possibility of scar contracture deformity. The surgical defect of the donor site could be closed primarily, as skin graft was not necessary. Therefore, the duration of aesthetic reconstruction and hospital stay decreased. Rehabilitation started sooner and patients were quite satisfied.

In conclusion, this modified S-shaped sural flap can be applied safely and reliably for reconstruction of foot and ankle defects with favorable aesthetic outcome. Regarding the simplicity of the dissection and flap transfer and low morbidity rate it seems that this modified technique could be a good choice in complex defects of the lower limb. However, it needs further investigations.
